# Quasi-critical fluctuations: a novel state of matter?

**DOI:** 10.1007/s11051-012-1407-2

**Published:** 2013-04-03

**Authors:** Erminald Bertel

**Affiliations:** Institute of Physical Chemistry, University of Innsbruck, Innrain 52a, 6020 Innsbruck, Austria

**Keywords:** Phase transitions, Fluctuations, Low-dimensional systems, Charge density wave, High-*T*_c_ superconductors

## Abstract

Quasi-critical fluctuations occur close to critical points or close to continuous phase transitions. In three-dimensional systems, precision tuning is required to access the fluctuation regime. Lowering the dimensionality enhances the parameter space for quasi-critical fluctuations considerably. This enables one to make use of novel properties emerging in fluctuating systems, such as giant susceptibilities, Casimir forces or novel quasi-particle interactions. Examples are discussed ranging from simple metal–adsorbate systems to unconventional superconductivity in iron-based superconductors.

## Introduction

Continuous phase transitions can be characterised by an order parameter which changes from zero above the critical temperature to a finite value below *T*_c_. The Landau theory of phase transitions allows a very succinct phenomenological description of such phase transitions. It uses an expansion of the Free Energy of the system in terms of powers of the order parameter:

1 $$ f\left( {x,T} \right) = f_{0} \left( {x,T} \right) - \left[
{hm\left( {x,T} \right)} \right] + \alpha \left( T \right)m^{2} \left( {x,T} \right) + \lambda m^{4}
\left( {x,T} \right) $$

Here, $$ f\left( {x,T} \right) $$ is the free-energy density, $$ m\left( {x,T} \right) $$ is the local order parameter, $$ \alpha \left( T \right) $$ is a coefficient, which depends linearly on $$ T $$: $$ \alpha \left( T \right) = \alpha_{0} \left( {T - T_{\text{c}} }
\right) $$, and $$ \lambda > 0 $$ is a constant, and *h* is an external field.

In field-free space, the expression contains only even powers of the order parameter, since the sign of the order parameter is irrelevant for the free energy. Consider for instance the order parameter of a ferromagnetic material, i.e. the local magnetisation. Obviously, the free energy depends only on the magnitude, not on the direction of the magnetisation. In the presence of an external field, however, the term given in square brackets in Eq. (1) has to be added to the expansion. It is linear in the order parameter, and hence changes sign, as either the order parameter or the external field are reversed.

Now we concentrate on the phase transition at $T_{\text{c}}$ in the absence of an external field. At $T_{\text{c}}$ both the first and the second derivative with respect to $m$ of the free energy are zero for $m = 0$. As a consequence, the order parameter is allowed to fluctuate around zero. Thus, the system is in a quite exceptional state, it exhibits critical fluctuations. This implies that the local order parameter varies in time and space from point to point. However it does so in a very peculiar manner, since the fluctuations are correlated. As $T$ approaches $T_{\text{c}}$ the correlation length diverges. If the phase with $m = 0$ is denoted as phase 1 and the phase with $m \ne 0$ as phase 2, then a diverging correlation length means that there are domains of either phase in the system which momentarily span the whole system. Moreover, at every moment, phase 2 is nucleating within phase 1 and vice versa. Thus, at a given time, phase 2 domains of every size are found in phase 1 and phase 1 domains of every size in phase 2. Consequently at $T_{\text{c}}$ the correlation length $\zeta$ is the only characteristic length scale in the system. With $\zeta$ diverging at $T_{\text{c}}$, the system is said to be scale free. The proliferation of phase boundaries in this state causes strong light scattering, the so-called critical opalescence.

## Novel properties

A material in a fluctuating state exhibits some more interesting properties. The fluctuation dissipation theorem states that the material’s response function $\chi \left( T \right)$ which characterises the response of a system to an external perturbation is proportional to the fluctuations of the order parameter:

2 $$ \chi \left( {\omega = 0,T} \right) = \frac{1}{{k_{B} T}}\left(
{\left\langle {m^{2} } \right\rangle - \left\langle m \right\rangle^{2} } \right)
$$

Thus, a fluctuating system is exceptionally sensitive against external perturbations. Consequently a material which can be kept in such a fluctuating state lends itself to applications in switching and sensing devices. The problem is of course that normally a precise fine-tuning of the thermodynamic parameters is required to keep the system close to the critical condition.

A further interesting aspect of fluctuations is the Casimir effect. If, for instance, point-, line-, or planar defects are immersed into the fluctuating system, the boundary conditions at the defects enforce a change of the fluctuation spectrum. Accordingly, the energy density between the defects is altered which results in an attractive or repulsive interaction between the defects depending on the boundary conditions (Hertlein et al. 2008). This is a generalised Casimir effect, the analogue to the interaction resulting from a modification of the vacuum fluctuation spectrum between to dielectric bodies.

**Fig. 1:**
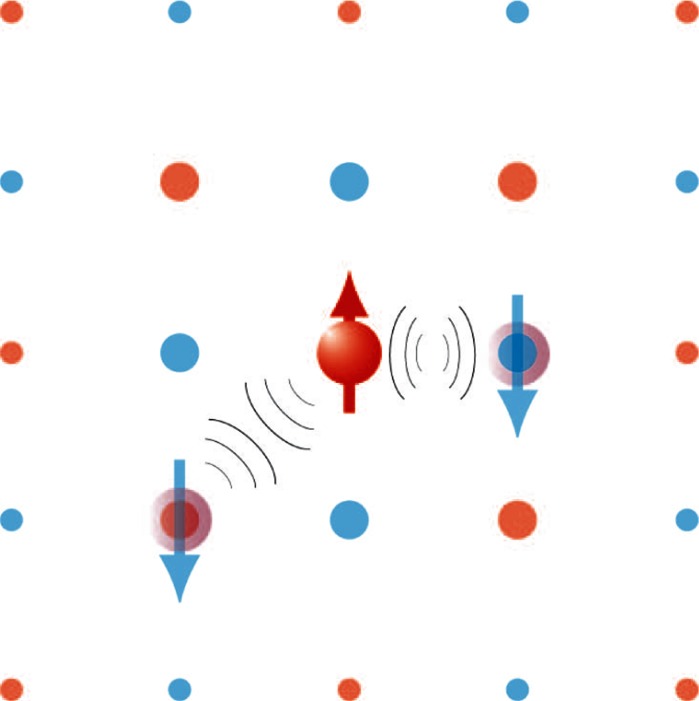
Schematic representation of spin-fluctuation mediated singlet pairing with d-wave symmetry. *Orange* (*blue*) sites represent preference for spin up (*down*) in an antiferromagnetic background. The interaction of a spin-up particle with a spin-down particle is attractive $$ \left( {\left( {\left( {} \right)} \right)} \right)
$$, if the latter occupies a nearest-neighbour (nn) site, while it is repulsive $$ \left. {\left. {\left. {} \right)} \right)} \right)\left( {\left(
{\left( {} \right.} \right.} \right. $$, if it occupies a nn site. (Color figure online)

**Fig. 2:**
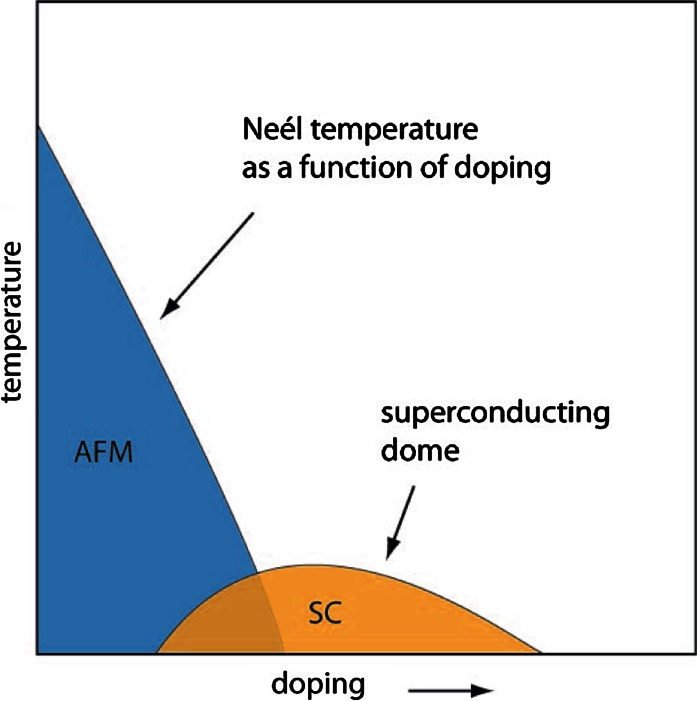
Schematic phase diagram of an unconventional superconductor

Even more importantly, fluctuations are believed to be responsible for the Cooper pairing interactions in the unconventional superconductors, namely the cuprates and the iron-based superconductors (Tôru and Kazuo 2003). Since in both cases the undoped parent compounds are antiferromagnetic, it is not surprising that spin density wave fluctuations are generally held responsible for the formation of the Cooper pairs. Figure 1 shows a cartoon of the interaction between charge carriers with opposite spin in an antiferromagnetically fluctuating background (Monthoux et al. 2007). Note that this type of interaction naturally explains the d-wave symmetry of the pairing interaction experimentally found in the cuprates. One should hasten to add that the subtle interactions governing the pairing and the formation of the Bose condensate require low temperatures. Hence only critical fluctuations at a phase boundary which in the phase diagram heads down to very low temperatures are relevant for superconductivity (see Fig. 2). If one extrapolates such a phase boundary through the superconducting dome, it is seen to cross the zero-temperature axis. At this point the fluctuations are obviously no longer thermally driven, but governed by quantum fluctuations. The property of materials in the vicinity of such a quantum phase transition are not yet very well understood (Sachdev 1999). Different scaling laws apply and it is for instance not clear, at what temperature above the quantum critical point the signature of quantum fluctuations disappears. It is often presumed that unconventional superconductivity is not only a consequence of quasi-critical fluctuations of, let’s say, the AF order parameter, but that it results from the system’s reluctance to enter that bizarre quantum regime close to the quantum critical point. Instead the system tilts over into a new phase, the superconducting phase. For the present purpose, it suffices to note that quasi-critical fluctuations along a phase boundary which heads towards the zero temperature axis as a function of some experimental parameter (doping, pressure, external field, etc.) are prone to produce new and exotic phases.

## Low-dimensional systems

As mentioned above, establishing quasi-critical fluctuations requires in general a precise fine-tuning to near a continuous phase transition or a critical point. In the following discussion, we concentrate on the temperature as the tuning parameter. In 3D, systems fluctuations are in general rapidly suppressed as the temperature deviates only by a few tenths of a degree from $T_{\text{c}}$. This problem can be solved by constructing low-dimensional systems. As the dimensionality is lowered, the temperature regime where appreciable fluctuations occur broadens considerably. Consider for instance a one-dimensional (1D) Ising chain of length *N* with exchange interaction *J* < 0, so that the groundstate is ferromagnetic. Flipping one of the spins in the chain changes the free energy *F*:

3 $$ \Updelta F = \Updelta U - T\Updelta S = \left| J \right| - k_{B}
T\ln N $$

with $\left| J \right|$ being the energy cost for the spin misalignment and $k_{B} \ln N$ the configurational entropy arising from the free choice on which of the *N* positions the spin is flipped. Obviously, for *T* > 0 the free energy is always lowered, provided that *N* is large enough. Hence the correlation length in such a system with discrete symmetry and solely nn interactions diverges only for $T \to 0$ K. Similarly, in a 2D system with continuous symmetry, e.g. a Heisenberg spin array, the correlation length will also diverge only for $T \to 0$ K (Mermin and Wagner 1966). Accordingly, fluctuations will prevail in such materials down to 0 K. The broad range where fluctuations are expected to dominate the behaviour of quasi-1D systems is also illustrated by the calculations of Anderson and co-workers (Lee et al. 1973) who show that in their system, the correlation length diverges only at about 20 % of the critical temperature $T_{\text{c}}$. The bottom line is that quasi-critical fluctuations are difficult to establish in a 3D material, but by engineering low-D materials it is possible to considerably enlarge the parameter space for such a fluctuating state and eventually harness the exotic properties associated with it for practical applications.

**Fig. 3:**
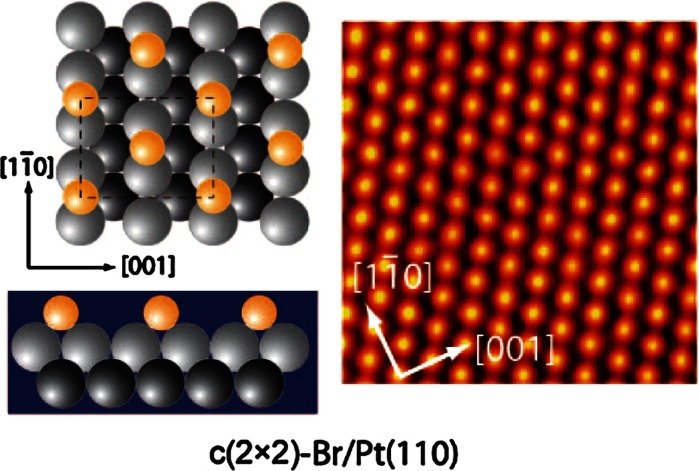
The c(2 × 2)-Br/Pt(110) surface: Ball model and STM topographic image (3.8 × 3.8 nm²) (Blum et al. 2002). *Gray* and *black balls* represent Pt atoms, *yellow balls* Br atoms. (Color figure online)

As a practical example we first consider a simple 2D system, i.e. an adsorbate on a transition metal. The model system chosen here is half a monolayer (ML) of Bromine on Pt(110). One ML is defined here as the surface atom density of the unreconstructed Pt(110) surface (9.2 × 10^18 ^m^−2^). Halogen adsorption lifts the (1 × 2)-missing-row reconstruction of the clean surface and at room temperature the Br forms a long-range-ordered c(2 × 2) structure as shown in Fig. 3 (Blum et al. 2002). This is a very common structure, because the quasi-hexagonal one packing the repulsive energy between the adatoms is minimised. Since the Pt(110) surface features close-packed atom rows with a nn distance of 0.277 nm, with the rows being separated by a lattice constant, i.e. 0.392 nm, the surface is strongly anisotropic. Actually, 1D electronic surface states are present and thus the surface may be considered as quasi-1D. Upon heating, a continuous disordering transition takes place.

**Fig. 4:**
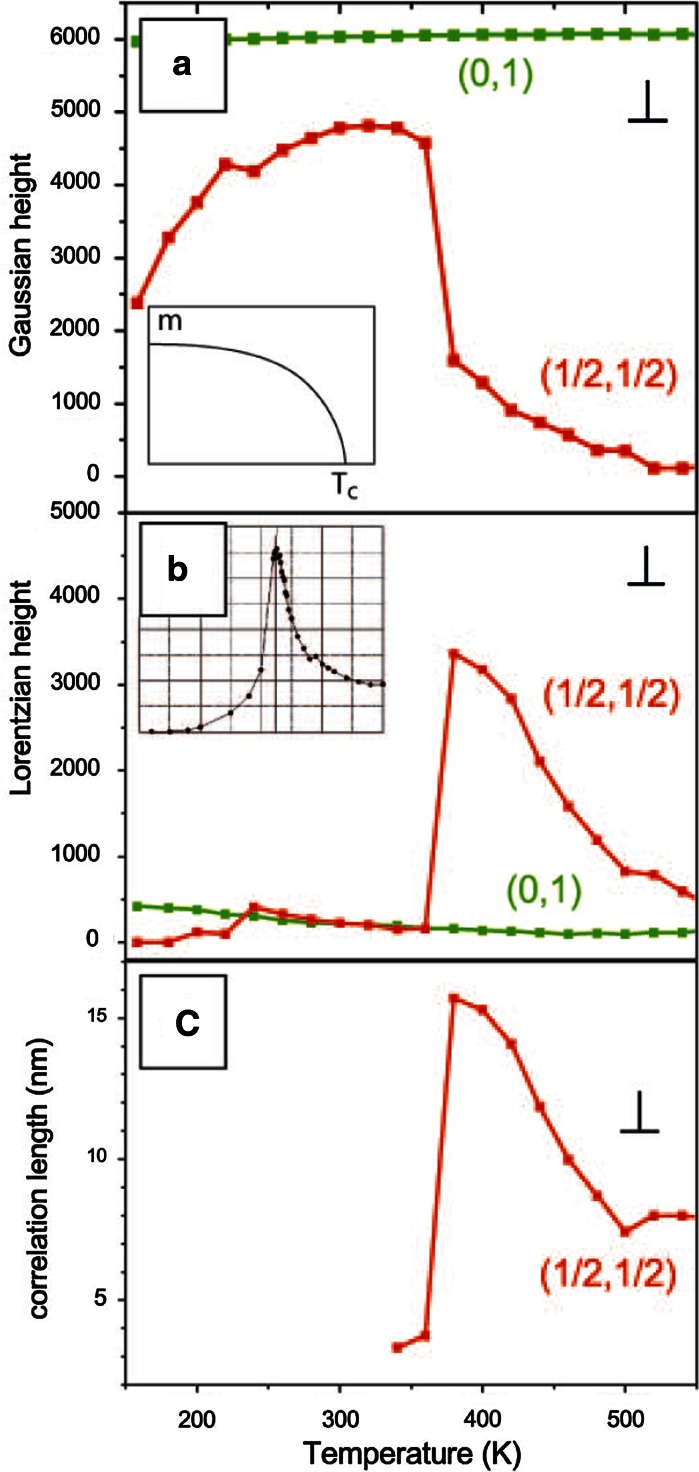
Spot profile analysis of the half-order (*red*) and an integer-order (*green*) LEED spot for the c(2 × 2)-Br/Pt(110) structure. The profile has been recorded in the [001] direction perpendicular to the close-packed Pt atom rows. **a** Gaussian component of the spot profile which is a measure of the order parameter (long-range order). *Insert* theoretical temperature dependence of order parameter near *T*_c_. **b** Lorentzian component which is a measure of the short-range order in a fluctuating system. *Insert* Monte-Carlo calculation of the Lorentzian amplitude at *T*_c_ in a finite 2D Ising system. **c** Correlation length of the fluctuations derived from the width of the Lorentzian component. (Color figure online)

The phase transition can be monitored by analysing the spot profile in low-energy electron diffraction (LEED). For a long-range-ordered system, the spot profile is a Gaussian and the height of the Gaussian serves as a measure of the order parameter. As shown in Fig. 4a, the Gaussian peak height drops precipitously as the disordering temperature at ~370 K is reached indicating the loss of long-range order. As a result of defect pinning at monatomic steps of the Pt(110) surface some residual order persists up to higher temperatures. Close to the transition temperature the peak profile is not a pure Gaussian. Due to the local fluctuating order a Lorentzian component appears and this is shown together with the correlation length derived from the width of the Lorentzian in Fig. 4b and c. From the temperature dependence of the Lorentzian peak height one can conclude that fluctuations in the system are prominent within a range of about 100 K around $T_{\text{c}}$. This illustrates the much larger range of fluctuations in low-D materials.

**Fig. 5:**
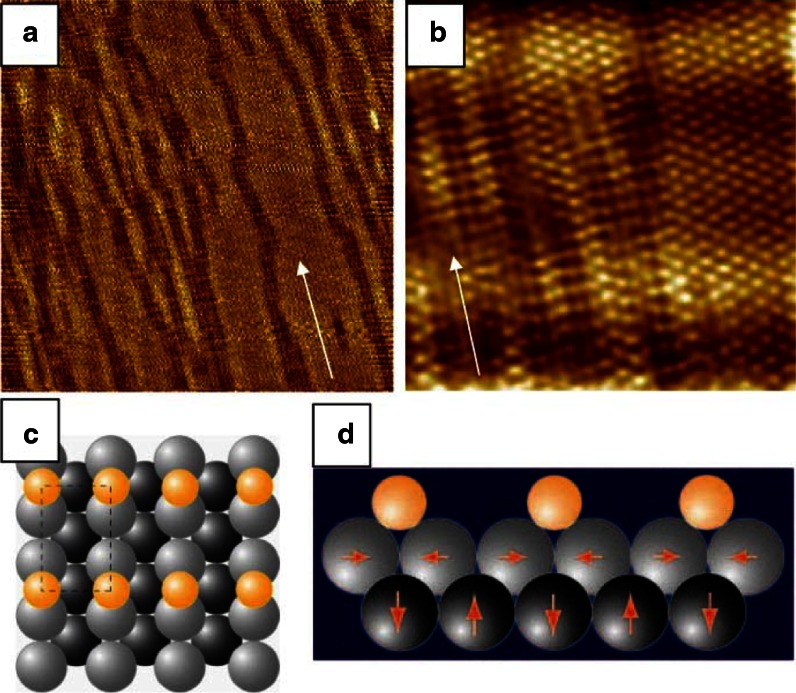
Phase transition c(2 × 2) → (2 × 1) occurring upon cooling. **a** The long-range ordered c(2 × 2) structure observed at room temperature decays into a striped pattern of bright and dark domains. **b** A close-up reveals the bright domains to be formed by c(2 × 2) and the dark domains by (2 × 1) order. **c** Ball model of the (2 × 1) structure: *grey balls* are substrate Pt atoms, *yellow balls* Br atoms. **d** Cross-section through the surface showing the buckling of the substrate. (Color figure online)

Conspicuously, however, Fig. 4a shows that long-range order is also lost upon cooling. This is surprising, since cooling of an ordered structure usually tends to improve the order. Scanning tunnelling microscopy (STM) topographic images recorded at 50 K (Fig. 5a, b) reveal why order is lost: Another phase appears, with Br atoms being arranged in a (2 × 1) unit cell. According to our DFT calculations this is the actual groundstate of the system in agreement with its appearance upon lowering the temperature. It is somewhat unexpected that the (2 × 1) structure is most stable, since it should exhibit a higher inter-adsorbate repulsive energy as compared to the c(2 × 2). Instead, heating the sample is needed for rearranging the Br atoms into the latter structure with extremely well-developed long-range order at room temperature. The presence of a second long-range-ordered structure for the same coverage at higher temperature is extremely rare in any adsorbate system. It requires this structure to have a much higher entropy than the groundstate. Since the long-range order excludes a substantial contribution from configurational entropy, the entropy gain in the adsorbate layer would have to arise from vibrational entropy. However the local bonding site is the same in both structures, so the vibrational entropy difference cannot explain this order–order transition. The solution to the problem lies in the substrate contribution (Cordin et al. 2010). While the Pt(110) surface is flat and essentially bulk truncated, there is a pronounced buckling present in the (1 × 2)-Br/Pt(110) surface (Cordin et al. 2012) as shown in Fig. 5c. This periodic lattice distortion (PLD) is associated with a periodic charge modulation (CDW) in the surface. The Br rows decorate the charge density maxima of the CDW.

Qualitatively, the observed phase transition can therefore be described as follows: To establish the low-*T* (2 × 1) structure an extra energy cost has to be spent on the inter-adsorbate repulsion and the distortion of the substrate. This extra energy, however, is over-compensated by an increased bonding strength of the Br to the substrate, as the latter offers more favourable bonding sites on the CDW maxima. Rising the temperature increases both, the lattice entropy and the electronic entropy in the substrate until the combined PLD/CDW ‘melts’. In other words, the periodic lattice and charge density modulation is increasingly blurred by thermal excitation of phonons and by excitation of electrons across the Peierls gap. As the PLD/CDW order parameter is thermally suppressed, the energy balance tilts in favour of the c(2 × 2) structure, since in the latter the inter-adsorbate repulsion is minimised. To substantiate this idea we investigate the individual free energy contributions in the system. The inter-adsorbate repulsion can be represented in an Ising-type model. To each of the adsorption sites $$ i $$ is assigned an occupation number $$ s_{i} = \pm {1 \mathord{\left/ {\vphantom {1 2}} \right. \kern-0pt}
2} $$, depending on whether it is occupied (+) or not (−). Since the Br–Br distance along the close-packed row direction (the [1 −1 0] direction) is the same in both structures, the difference in repulsive energies arises solely from the difference in the occupation of nn sites in the [001] direction. Accordingly, in a (minimal) 1D model the repulsive energy can be represented by a term $$ \sum\limits_{i} {Js_{i} s_{i + 1} } $$, where $$ i $$ counts the adsorption sites in [001] direction. For the c(2 × 2) structure, the occupation numbers change sign from place to place, and therefore the contribution to the free energy is negative. In the (2 × 1) structure in contrast, all $$ s_{i} $$ are positive and a positive contribution to the free energy is obtained. The substrate contribution is provided by the usual Landau expansion of the free energy in terms of powers of the order parameter, here the amplitude of the PLD/CDW. Finally we need to represent the adsorbate coupling to the substrate CDW. To this end we introduce a coupling term $$ \sum\limits_{i} {gm\left( {s_{i} + s_{i + 1} } \right)}
$$. For $$ g < 0 $$ and $$ m \ne 0 $$ , this term lowers the total free energy, if the adsorbate forms a (2 × 1) phase, but it is zero in the c(2 × 2) phase and also, if $$ m = 0 $$, i.e. if the substrate PLD/CDW is suppressed. Thus, one obtains in total (ignoring a constant contribution $$ F_{0} $$ to the free energy):

4 $$ F = \sum\limits_{i} {Js_{i} s_{i + 1} + \sum\limits_{i} {gm\left(
{s_{i} + s_{i + 1} } \right)} } + \alpha \left( T \right)m^{2} + \lambda m^{4} $$

The resulting free energy surface as a function of temperature and order parameter is discussed in detail in ref (Cordin et al. 2012). Obviously, on the flat substrate $m = 0$ and owing to the first term the free energy is minimised, if $s_{i} = - s_{i + 1}$. This is the signature of the c(2 × 2) structure. If the substrate is buckled $\left( {m \ne 0} \right)$, then the coupling term becomes effective. For strong enough coupling, it will outweigh the first term and thus favour the (2 × 1) structure. Note that the second term has the form of an external field contribution to the free energy (compare Eq. 1). Thus, it stabilises a finite value of the order parameter even for $T > T_{\text{c}}$. Actually, $T_{\text{c}}$ could even be negative implying that the PLD/CDW is unstable at all temperatures on the clean surface. Nevertheless, on the adsorbate covered surface an order parameter $m \ne 0$ could persist up to some finite temperature due to the stabilisation of the PLD/CDW as the adsorbate locks into the CDW fluctuations. The adsorbate freezes them into a (more or less) static PLD/CDW phase in a bootstrap type mechanism. The presence of the coupling term in Eq. (4) renders the phase transition weakly first order. The stability of the system then depends on the barrier in the free-energy surface at a given temperature. Actually, in the present system there are still c(2 × 2) ⟷ (2 × 1) fluctuations present at $T = 50$ K, albeit on a time scale of several seconds.

As pointed out before, such a type of phase transition is extremely rare in adsorbate systems. Obviously it requires an instability of the substrate to CDW fluctuations. For Pt, this is not too surprising, as a strong Kohn anomaly has been observed even in the bulk (Tsunoda 2011). But is there any significance beyond this system? The answer is yes.

## Application to iron-based superconductors

Very recently a discussion arose about the origin of surface structures observed on the cleavage planes of 122 Fe-based superconductors AFe_2_As_2_, where 122 refers to the chemical composition and A is an earth alkali metal (Ba, Sr, Ca) (Hoffman 2011). On these cleavage planes c(2 × 2) and (2 × 1) structures where observed, with the former prevailing after room temperature cleavage of BaFe_2_As_2_ and SrFe_2_As_2_, while the latter was found after low-temperature cleavage. On CaFe_2_As_2_ the (2 × 1) structure was stable also after room temperature cleavage. Long-range ordered domains were often found to co-exist with disordered areas. The results were similar for the undoped parent compounds and the doped superconductors, with a possible dependence of the relative stability of the two structures on doping.

**Fig. 6:**
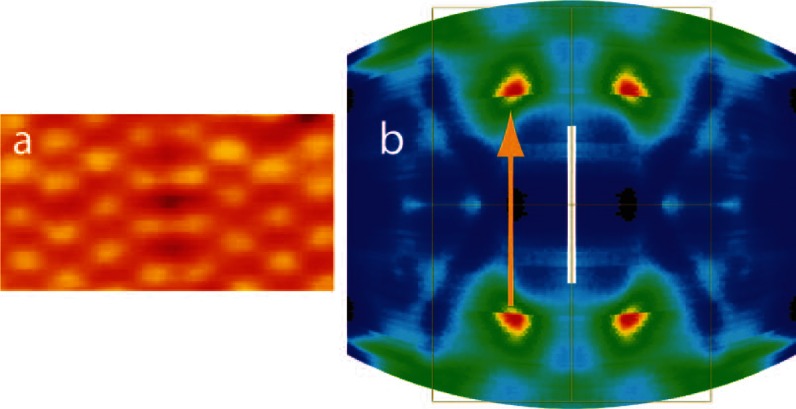
**a** Coexistence of c(2 × 2) and (2 × 1) domains showing a striking similarity to surface structures observed on SrFe_2_As_2_ (Niestemski et al. 2009). **b** Fermi surface map of Pt(110) recorded in angle-resolved photoemission spectroscopy. The thin *rectangle* delineates the surface Brillouin zone. *Blue* (*red*) denotes low (high) photoemission intensity. The *orange arrow* indicates a wave vector *q* of excitations between two points of high density of states at the Fermi level. (Color figure online)

The interpretation of the observed structures was subject to controversy. While some groups favoured an explanation in terms of earth alkaline metal adsorbate superstructures (Boyer et al. 2008; Yin et al. 2009; Hsieh et al. 2008; Massee et al. 2009; Zhang et al. 2010), other groups proposed reconstructions of the arsenic top-layer (Niestemski et al. 2009; Nascimento et al. 2009; Li et al. 2010). Here we take side with the adsorbate superstructure interpretation. This is motivated by (i) the striking similarity of STM topographs obtained on Br/Pt(110) as compared to results from AFe_2_As_2_ cleavage planes [see Figs. 6a, 2h in ref (Niestemski et al. 2009)], (ii) the similar relative stability of the long-range ordered structures as a function of temperature and composition and (iii) similar structures in the Fermi surface mapping obtained by angle-resolved photoemission spectroscopy (ARPES).

Argument (i) is perhaps somewhat phenomenological, but it would be surprising, if a reconstruction of a bare surface would produce not only a similar contrast, but also identical domain structures of coexisting adsorbate phases with a coverage of 0.5 ML.

Argument (ii) deserves a more detailed consideration. The existence of two different long-range ordered structures depending solely on temperature is not trivial to explain, neither in the bare-surface, nor in the adsorbate-on-surface model. In both cases, the substrate has to co-operate in the phase transition delivering a substantial entropy contribution in the high-temperature structure. As pointed out above, the entropy difference could result from the ‘melting’ of a CDW in the substrate. This possibility has in fact been considered by Niestemski et al. (2009), but was discarded, because they did not observe a contrast inversion in the STM image, as the bias was reversed. This conclusion, however, is not justified, if the CDW is decorated by an adsorbate. Moreover, on a bare surface the melting of a CDW above the critical temperature should result in a (1 × 1) structure rather than a c(2 × 2) as it is found in the present case. Further support for the adsorbate-on surface hypothesis derives from theoretical work by Gao et al. (2010). Their ab initio DFT calculations showed the surface with 0.5 ML earth-alkaline metal coverage to be the energetically preferred one. As to the superstructure, the c(2 × 2)-A structure was found to be the most stable one for A = Ba, to be marginally stable for A = Sr, and to be unstable with respect to the (2 × 1)-A structure for A = Ca. Of course, DFT total energy calculations yield the groundstate for *T* = 0 K and as such are not able to predict or explain phase transitions. As judged from experiment, the equilibrium groundstate is more likely the (2 × 1) state for all three cases, but the trend in relative stability is apparently correctly represented in the DFT calculations (Cordin et al. 2012; Gao et al. 2010).

Our extended Landau model Eq. (4) yields an explanation for the phase transition as well as for the relative stability: since the first term in Eq. (4) representing the inter-adsorbate repulsion favours the c(2 × 2) structure and since the repulsive energy *J* is expected to be the largest for Ba and the smallest for Ca, the c(2 × 2) structure should indeed be the most stable for BaFe_2_As_2_, as predicted by Gao et al. (2010). The explanation of the different structures as a function of temperature in the present model is based on the assumption of a CDW instability in the substrates, viz. the AsFe_2_As sandwiches.

This leads to argument (iii) from above: in Pt(110), the CDW instability was attributed to a ‘nesting’ vector between two points of high density-of-states (DOS) at the Fermi level (see Fig. 6b). A similar nesting condition is not anticipated for the AFe_2_As_2_ compounds, at least not in many of the model band structures on which the analysis of antiferromagnetic correlations in the 122 compounds is usually based. Note that the ‘nesting’ vector underlying the CDW correlations postulated in the present model includes an angle of 45° with the nesting vector which is held responsible for the antiferromagnetic instability. Recent ARPES results, however, are at variance with the band topology at the surface Brillouin boundaries (SBZ) proposed in some simplified band structure models (Zabolotnyy et al. 2009; Kondo et al. 2010; de Jong et al. 2010). Points of high ARPES intensity and consequently high DOS are found at the Fermi level which resemble closely the ones seen in the Fermi surface map of Pt(110) shown in Fig. 6b (Cordin et al. 2012). Neither in Pt(110) nor in the 122 Fe arsenides do the connecting vectors precisely match half a reciprocal lattice vector as expected for a CDW of period 2. If this were the case, the result would presumably be a static CDW rather than CDW fluctuations. One should also be careful in applying nesting arguments too rigidly as pointed out by Mazin and coworkers (Johannes and Mazin 2008). Usually, the structures in the response function $\chi \left( q \right)$ caused by Fermi surface nesting are not sharply peaked, since they result from integration over a finite energy interval around E_F_ and in addition are weighted by the electron–phonon coupling.

The present model attributes the (2 × 1) phase to a CDW in the AsFe_2_As sandwich layer which is stabilised in a bootstrap mechanism by the earth alkaline metal atoms. In the bulk compound, however, this mechanism cannot operate, since there is a full A layer separating the AsFe_2_As sandwiches. Thus, instead of a static CDW, only charge density fluctuations are expected. The corresponding wave vector is oriented in the real space direction of the pairing interaction (Zhai et al. 2009). It is conceivable that a surface CDW stabilising the (2 × 1) structure could also originate from fluctuating orbital order (Kontani and Onari 2010) with ${\mathbf{q}} = \left( {\pi ,0} \right)$ (Zhou et al. 2011).

## Conclusion

Tuning materials into a quasi-critical fluctuation regime offers the opportunity to make use of exotic properties associated with such a fluctuating state. Among these properties are a strong response to external perturbations and novel types of interactions, such as the Casimir force or Cooper pairing. The parameter range in which fluctuations persist is considerably enhanced in low-dimensional systems. Hence, precision tuning can be avoided, if low-dimensional systems are constructed. As an example, a metal–adsorbate system is analysed, in which charge-density fluctuations cause an unconventional phase transition. The phase transition is modelled in the spirit of Landau theory by expanding the free energy in terms of a CDW order parameter, but also adding terms representing the inter-adsorbate repulsion and the adsorbate–substrate coupling. Transferring the same model to the controversially discussed surface structures observed on 122 Fe-based superconductors a consistent explanation of both, the temperature dependence and the relative stability of the structures as a function of chemical composition is reached. As a consequence, it is suggested that CDW (or eventually orbital order) fluctuations are present in these compounds with a wave vector differing by an angular offset of 45° from that of the AFM–SDW fluctuations. On the one hand this underlines the pivotal role of fluctuations in unconventional superconductors, on the other hand it illustrates the rich phenomenology accessible by steering different order parameters into the fluctuation regime.
